# Hepatorenal Protective Effects of Hydroalcoholic Extract of *Solidago canadensis* L. against Paracetamol-Induced Toxicity in Mice

**DOI:** 10.1155/2022/9091605

**Published:** 2022-12-17

**Authors:** Omid Rahimi, Nilufar Asadi Louie, Alireza Salehi, Firouz Faed Maleki

**Affiliations:** ^1^Department of Pharmacology, Babol Branch, Islamic Azad University, Babol, Iran; ^2^Department of Pathology, Babol Branch, Islamic Azad University, Babol, Iran

## Abstract

Paracetamol (AKA acetaminophen) is a widely used drug and is used for mild to moderate pains, such as mild osteoarthritis, toothache, headache, and pain caused by minimally invasive surgeries. Despite being a harmless drug in lower doses, acetaminophen can be toxic to the liver and kidneys if overdosed and even results in death. In this study, the therapeutic effects of *Solidago canadensis* L. extract (SCE) were investigated. 48 adult male Swiss albino mice (20–30 grams) were randomly divided into six groups of 8. The control group was gavaged with normal saline every 12 hours for 6 days. The second group received paracetamol at a 500 mg/kg intraperitoneally (i.p) dose on the sixth day. The third, fourth, and fifth groups were gavaged doses of 125, 250, and 500 mg/kg of SCE every 12 hours for six days, respectively, and on the sixth day, we received paracetamol at a dose of 500 mg/kg i.p. The sixth group only received SCE every 12 hours at a dose of 1000 mg/kg via gavaging for six days. On the seventh day (24 hours after paracetamol injection), blood samples were collected to measure the serum level of creatinine, uric acid, blood urea nitrogen (BUN), total protein, albumin, alanine transaminase (ALT), aspartate transaminase (AST), alkaline phosphatase (ALP), and total and direct bilirubin, and liver and kidney tissues were also sampled for histopathological examination. It was observed that paracetamol caused a considerable increase in the ALT, AST, ALP, uric Acid, and BUN levels (*P* < 0.01), while those in SCE-treated groups were significantly lower. In addition, various lesions in the paracetamol group were observed, while in the SCE-receiving groups, receiving prophylactic SCE inhibited the high-intense lesions such as the infiltration of inflammatory cells, hyperemia, and vacuolar degeneration, which decreased significantly in the control group in comparison with that of the paracetamol group (*P* < 0.05). In conclusion, SCE can have substantial protective effects against paracetamol's hepatorenal toxicity.

## 1. Introduction

Paracetamol, chemically known as acetaminophen (4′-hydroxyacetanilide, N-acetylp-aminophenol, PAR), is the most common over-the-counter analgesic and antipyretic drug with mild anti-inflammatory effects, which was first described in 1893 by Von Mering [1, 2]. This drug can be used alone or in combination with other drugs with more than 200 formulas for the symptomatic treatment of pain, fever, cough, and cold [3, 4]. One of the most common causes of renal failure and hepatic necrosis in humans and animals is the excessive use of paracetamol [5]. The main reasons for this can be easy access to this medication as well as limited knowledge of consumers and some physicians about the side effects of overuse of this medication. If used in therapeutic doses, paracetamol is a relatively safe drug with limited side effects, but in higher doses, it can be potentially hepatotoxic [6–8] Although nephrotoxicity is less likely to occur, acute renal failure and renal tubular damage have been reported in patients with major liver injury and who are more severely poisoned [9]. Due to the lack of an effective treatment for hepatic diseases and the overuse of such synthetic medications, it has become critical to find natural treatments with fewer side effects. Hence, the present study was planned to determine the protective effect of *Solidago canadensis* extract on paracetamol-induced hepatic and renal toxicity in mice. Moreover, it was observed that cytokines play a vital role in increasing paracetamol-induced hepatotoxicity by utilizing and activating neutrophils and macrophages in the liver. Activated macrophages and neutrophils produce inflammatory and oxidative stress molecules, such as reactive oxygen species (ROS), leukotrienes, and cytokines, leading to more inflammation and further damage to the liver [7]. N-Deacetylase, prostaglandin endoperoxide synthase (PGES), and cytochrome P450 enzymes were tested for paracetamol-dependent toxicity in one or more species. Acute doses of paracetamol can lead to necrosis of the cortex and kidney [10]. Although hepatotoxicity is generally accepted, renal toxicity is less common in paracetamol toxicity. Usually, renal toxicity occurs when hepatotoxicity has already happened. With this in mind, preventing hepatotoxicity can be considered one of the most crucial ways to stop renal toxicity from happening [7].

The use of medicinal plants is a very rich tradition among people around the world mainly because of the flavonoid and phenolic compounds [11] in these plants and its biological properties, antioxidants, and the ability to scavenge free radicals. According to the World Health Organization, 80% of the world's population used herbs as their main means of health care. Today, in the field of pharmacology, many modern medicines are made in a way that was used directly in the past.

The genus *Solidago* is considered one of the largest genera of the Asteraceae family, including more than 120 species, most of which are found in North and South America. This genus includes many species such as *S. virguara*, *S. gigantean*, and *S. canadensis*. In addition to its therapeutic and anti-inflammatory properties, it is also a fine diuretic. It contains a substantial amount of flavonoids, which has strong antioxidant potential to this plant [12]. According to the European Pharmacopoeia, flavonoids are a quality marker of *Solidaginis herba*. Quercetin, kaempferol, isorhamnetin, and rhamnetin-3-O-glycosides are major flavonoids. Caffeoyl quinic acids (chlorogenic acid and others) are the second class of phenolic compounds [13]. Flavonoids, especially quercetin and derivates, inhibit the neutral endopeptidase enzyme, which is responsible for the interaction of the atrial natriuretic peptide and thus regulates the formation of urine via the excretion of sodium ions. This can be interpreted as the basis of enhanced urinary flow therapy [14, 15].

It was previously shown that *S. canadensis's* flavonoids, saponins, and caffeic acid esters can reduce the activity of leukocyte elastase, which is a protease involved in the development of inflammation [16]. *S. canadensis*'s parts are traditionally used as medicinal herbal tea with a diuretic activity in order to treat the urinary tract, kidney, and prostate stones. In traditional medicine, this plant is called a blood purifier for the eczema, gout, arthritis, rheumatism, and other skin disorders [16]. In this study, the effect of *Solidago canadensis* L. extract (SCE) on paracetamol-induced toxicity was investigated.

## 2. Methods and Materials

### 2.1. Drugs and Chemicals

The extract of *S. Canadensis* was purchased from Barij Essential Pharmaceutical Company, Kashan, Iran (batch number: 61310013, serial number: 1990940). The paracetamol was purchased from DarouPakhsh Pharmaceutical MFG Company.

### 2.2. Animals and Monitoring

48 male Swiss albino mice (weight 20–30 grams) were purchased from the Pasteur Institute of Iran. All animals were maintained in an air-conditioned room (21 ± 4°C) with a relative humidity of 65 ± 5%, alternating 12-hourlight-dark cycle, and were fed (ad libitum) with a certified pellet rodent diet (behparvarCo, Tehran, Iran) and free access to water. They were acclimatized in our animal housing center for at least 48 hours before the experiment [17].

All experimental procedures were approved by the research ethics committees following the accepted principles of working with laboratory animals and the instructions of the National Institutes of Health at all stages of the experiment (IR.IAU.BABOL.REC.1400.085 and IR.IAU.BABOL.REC.1400.089).

### 2.3. Treatment Groups

The animals were divided randomly into six groups (number of mice per group = 8) as follows:

 
*Group 1.* The control group: normal saline was administered to mice every 12 hours via gavage for 6 days
 
*Group 2.* The paracetamol group: Mice received the paracetamol intraperitoneally (IP) 500 mg/kg only on the 6th day [18]
 
*Groups 3–5.* The treatment groups: Mice received SCE orally every 12 hours (125, 250, and 500 mg/kg, respectively) for 6 days, and on the sixth day, they received paracetamol at a dose of 500 mg/kg intraperitoneally
 
*Group 6.* The SCE group: Mice received only the SCE for 6 days at a dose of 1000 mg/kg every 12 hours

### 2.4. Biochemicals and Histopathological Parameters

24 hours after paracetamol administration, mice were anesthetized with a combination of ketamine and xylazine [19], and then, the blood samples were collected via cardiac puncture to evaluate the serum markers such as ALT, AST, ALP, BUN, creatinine, uric acid, total protein, albumin, and direct and total bilirubin markers by an autoanalyzer (Parsian Az Teb, Sphera machine). To do so, blood samples were centrifuged for 10 minutes at 1500 RPM, and serum was collected from the upper part of the tubes. For histopathological examination, the liver and kidney tissues of all mice were immediately washed with phosphate buffer (pH 7.4) for the complete removal of any blood stains and clots. After that, the tissues were placed in a 10% formalin buffer solution for 24 hours to be fixated. After fixation of tissues, they were processed and cut into five microslides and then were stained with the H&E staining. Eventually, the tissues were evaluated descriptively under an Olympus CX23 light microscope [20].

### 2.5. Statistical Analysis

Parametric tests were performed, taking into account variable characteristics. The data obtained from the blood samples evaluations were analyzed by ANOVA with repeated measures, followed by Tukey's post hoc test. The values were presented as the mean ± standard error of the mean (SE). The minimum significant level was set to *P* < 0.05 [21], and higher significance levels were used appropriately [11,22]. Also, the severity of lesions in the histopathological examination was defined as no lesion (-), mild lesions (*∗*), moderate lesions (^∗∗^), and severe lesions (^∗∗∗^) [23].

## 3. Results

### 3.1. Biochemical Changes

**Figure 1:**
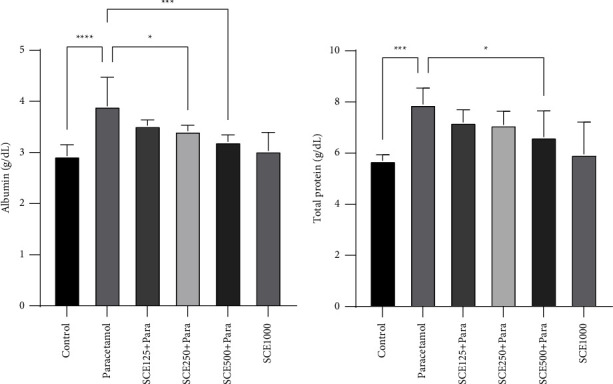
Comparison of total protein and albumin among all groups. ^*∗*^*P* < 0.05, ^*∗∗∗*^*P* < 0.001, and ^*∗∗∗∗*^*P* < 0.0001: significant compared to the paracetamol group; number of mice per group = 8.

The paracetamol-induced mice compared to the control group showed a noticeable surge in their serum total protein and albumin levels from 5.7 and 2.93 g/dL to 7.9 and 3.9 g/dL, respectively (*P* < 0.001 and *P* < 0.0001, respectively). Meanwhile, among the SCE-treated groups, total protein did not rise remarkably at the dose of 500 mg/kg (*P* < 0.05) and Alb was at its lowest between the doses of 250 and 500 mg/kg compared with the paracetamol group (*P* < 0.05 and *P* < 0.001, respectively) (Figure 1).

**Figure 2:**
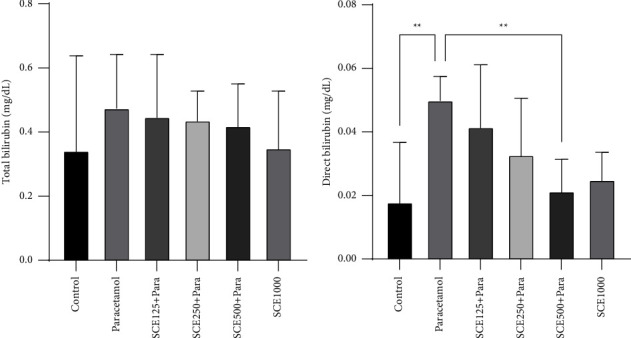
Comparison of total bilirubin and direct bilirubin among all groups. ^*∗∗*^*P* < 0.001: significant compared to the paracetamol group; number of mice per group = 8.

After administering paracetamol, it was observed that the level of total bilirubin had no significant change, but the amount of direct bilirubin surged significantly (*P* < 0.01) from 0.05 mg/dL in the paracetamol group as opposed to the control group at 0.02 mg/dL. Similarly, treatment with SCE at a dose of 500 mg/kg reduced direct bilirubin levels significantly (*P* < 0.01) (Figure 2).

**Figure 3:**
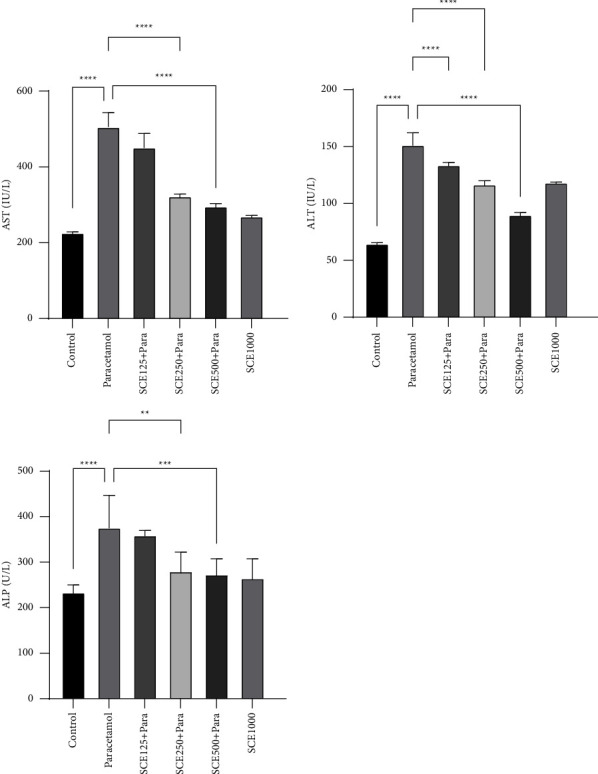
Comparison of liver biochemical markers among all groups. ^*∗∗*^*P* < 0.01, ^*∗∗∗*^*P* < 0.001, and ^*∗∗∗∗*^*P* < 0.0001: significant compared to the paracetamol group; number of mice per group = 8.

Regarding the liver markers, it was observed that after the administration of paracetamol, the level of all three ALT, AST, and ALP increased significantly (*P* < 0.0001) from 64.71, 226.8, and 234.1 IU/L in the control group to 151.7, 506.1, and 376.3 IU/L in the paracetamol group, respectively. However, both ALT and AST levels in all three SCE-receiving groups were significantly (*P* < 0.0001) reduced compared to the paracetamol group, and the ALP reduction was only significant in the higher-doses groups of SCE250 and SCE500 (*P* < 0.01 and *P* < 0.001, respectively) (Figure 3).

**Figure 4:**
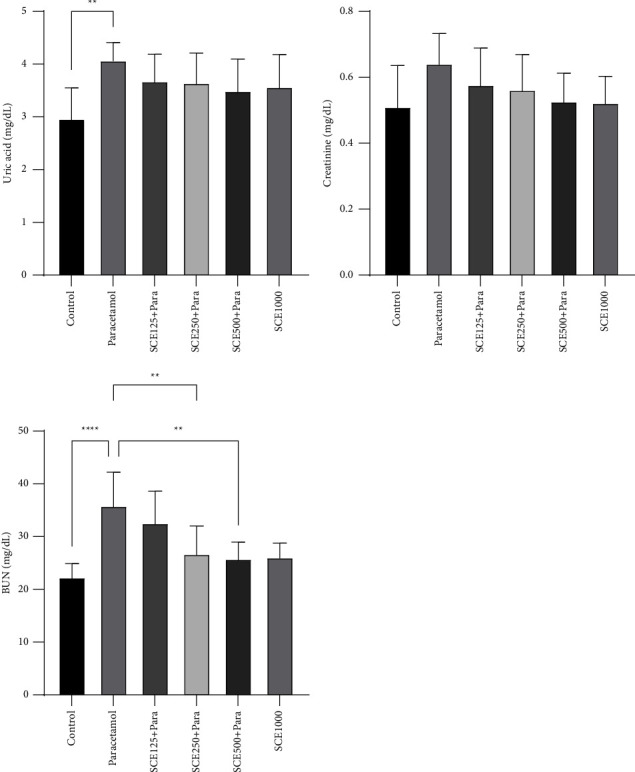
Comparison of kidney biochemical markers among all groups. ^*∗∗*^*P* < 0.01 and ^*∗∗∗∗*^*P* < 0.0001: significant compared to the paracetamol group; number of mice per group = 8.

While studying the renal parameters, it was observed that the creatinine level decreased in all SCE-treating groups compared to that of the paracetamol group. In the case of the uric acid, despite a significant (*P* < 0.01) increase from 2.97 to 4.08 mg/dL in the paracetamol and control groups, no significant decrease was seen in the SCE-treating groups. Finally, the BUN level increased significantly (*P* < 0.0001), after the paracetamol administration, from 22.36 to 35.88 mg/dL compared to that of the control group, and also, a significant (*P* < 0.01) decrease was observed in the SCE250 and SCE500 treatment groups compared to that of the paracetamol group (Figure 4).

### 3.2. Morphological and Pathological Changes

**Figure 5:**
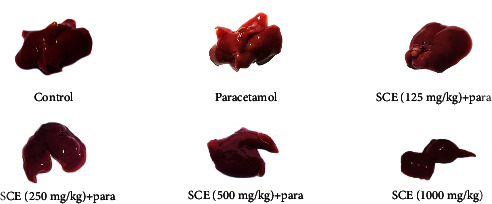
Morphological comparison between parenchyma of different groups in the liver.

**Figure 6:**
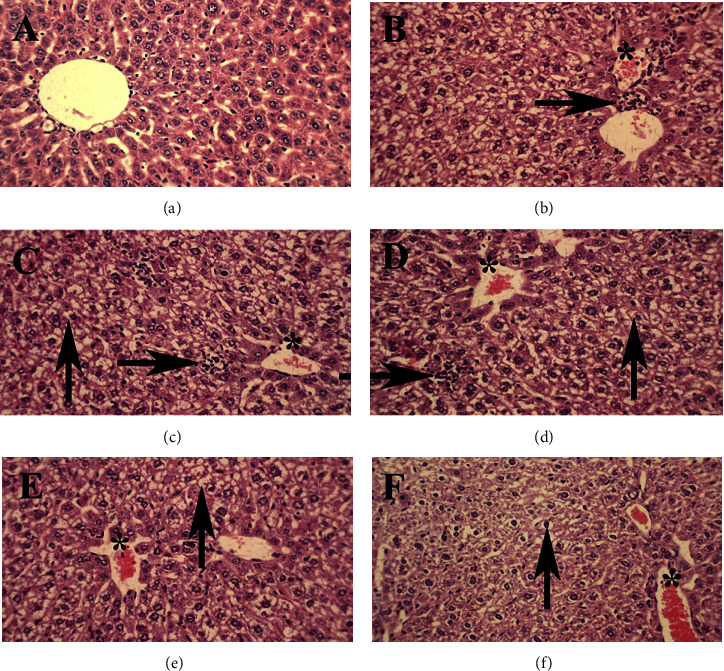
Liver: (a) Control group, normal tissue conditions, (b) paracetamol group, (c) SCE125 + para group, (d) SCE250 + para group, (e) SCE500 + para group, and (f) SCE1000 group. The hyperemia (star), infiltration of inflammatory cells (left arrow), and vacuole degeneration (up arrow) are illustrated. X40 magnification, H&E staining.

In the paracetamol group, the liver tissue went pale and was morphologically brighter and softer than those of other groups (Figure 5). In addition, the histological examination showed that after administrating paracetamol at the dose of 500 mg/kg, the hyperemia and vacuolar degeneration were significantly increased, and the presence of inflammatory cells in the liver parenchyma substantially rose. Generally, in the treatment groups, lesions were reduced. Moreover, in the SCE125 and SCE250, the severity of the lesions was greatly suppressed (*P* < 0.05), and in the SCE500 group, hyperemia significantly decreased (*P* < 0.01). Also, the number of inflammatory cells in the liver tissue of SCE500 group was insignificant compared to that of the control group (Table 1) (Figure 6).

**Figure 7:**
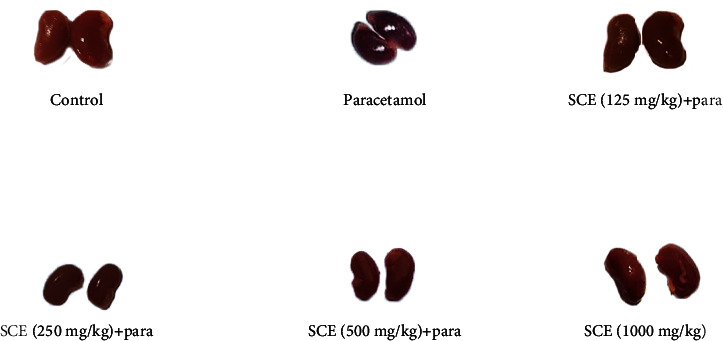
Morphological comparison between parenchyma of different groups in the kidney.

**Figure 8:**
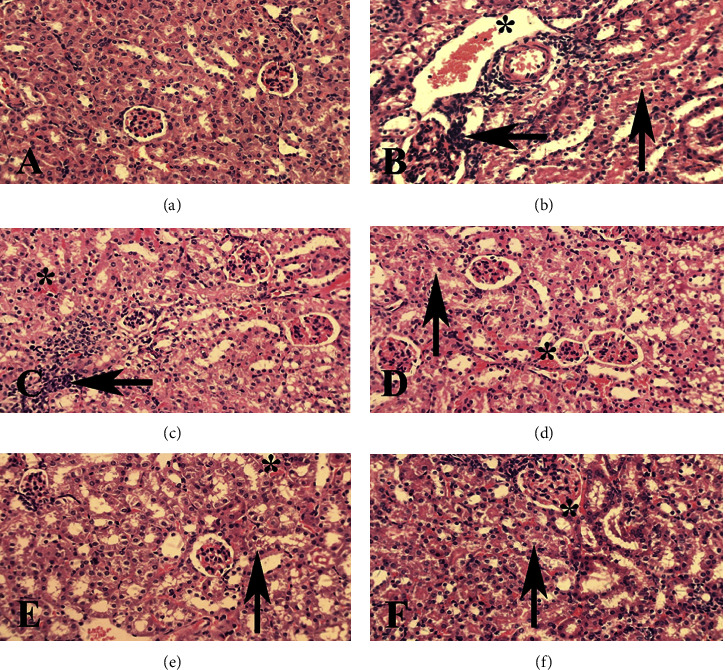
Kidney: (a) Control group, normal tissue conditions, (b) paracetamol group, (c) SCE125 + para group, (d) SCE250 + para group, (e) SCE500 + para group, and (f) SCE1000 group. The hyperemia (star), infiltration of inflammatory cells (left arrow), and vacuole degeneration (up arrow) are illustrated. X40 magnification, H&E staining.

The kidney tissue of the paracetamol group was moderately more bluish compared to that in the other groups (Figure 7). Histological examination of renal tissues showed that after paracetamol administration, the rate of hyperemia and vacuolar degeneration were significantly increased in the renal parenchyma, and the inflammatory cells penetrated the tissue (Table 2). In the SCE-receiving groups, the number of lesions was decreased, so that in the treatment group with SCE125 and SCE250, the severity of hyperemia, inflammation, and vacuolar degeneration gets significantly reduced (*P* < 0.05), and in the SCE500 group, the condition was even better, meaning that due to an insignificant number of inflammatory cells observed in the kidney tissue, the situation was predominantly better (*P* < 0.01) than other groups because no inflammatory cell was seen in the kidney tissue (Figure 8).

## 4. Discussion

The present study tried to reveal the preventive effect of SCE in paracetamol-induced hepatorenal toxicity in mice. Overall, SCE treatment reduced both biomarkers associated with paracetamol-induced toxicity and tissue damage in the liver and kidneys.

Paracetamol has been used in many articles to induce hepatic and renal toxicity [24–26], and in this study, agreeing with Nithianantham et al. [27] and Mani and Singh's [28] studies, ALP, ALT, AST, bilirubin, creatinine, urea, and BUN indices were elevated in mice. According to an earlier study, it seemed after paracetamol was metabolized to sulfate and glucuronide (a pharmacologically inactive form of paracetamol), more than 90% of this substance was excreted by the kidneys. However, less than 5% was converted to N-acetyl-para-benzoquinone imine (NAPQI) by cytochrome P450, which is highly reactive and unstable [29]. By binding to mitochondrial proteins, NAPQI causes mitochondrial dysfunction and DNA damage, which eventually leads to the death of hepatocytes [30, 31]. So, both the liver and kidneys need to be examined in this research. Keeping in mind that cellular damage was primarily limited to the proximal tube and a significant reduction in the glomerular filtration rate [32]. Renal microsomes, like their hepatic counterparts, oxidize paracetamol to an aryl mediator through a P450-dependent mechanism. As a result, at least parts of acute paracetamol-dependent renal dysfunction are believed to be based on a biochemical mechanism similar to that of the liver [1]. In addition, paracetamol is deacetylated to aminophenol in hepatic and renal microsomes and cytosols [33]. Therefore, in the chronic consumption of low-dose paracetamol, kidneys may be more vulnerable than the liver.

In the present study, paracetamol at a dose of 500 mg/kg was administrated to induce hepatorenal toxicity [34]. In order to evaluate the protective properties against paracetamol, SCE with three therapeutic doses of 125, 250, and 500 mg/kg was employed to ensure different levels of therapeutic potentials and probable toxicity. Overall, SCE has been shown to have high levels of flavonoids, antioxidants, and natural anti-inflammatory substances, and since these compounds can exhibit protective properties, they can be served as an ideal alternative to prevent paracetamol-induced toxicity. In various studies, different substances and flavonoids that make up the *Solidago canadensis* plant have been reported [35–43], especially quercetin as the dominant constituents and other flavonoids, namely, kaempferol, isorhamnetin, and caffeic phenyl acid as second-class components [13]. It has also been claimed that flavonoids of different *Solidago* species have different functions in diuretic properties [44–46], which is in line with Apati et al.'s [47] study based specifically on flavonoids which explained that the presence of quercetin and its derivatives was the reason for the increase in urine output. Several other studies also confirmed the diuretic properties of SCE and certain herbs of which flavonoids are their main components [48–50]. Finally, to observe any possible side effects caused by SCE or any overdose, a group with a daily dose of 1000 mg/kg was considered. However, no significant damage from SCE at a dose of 1000 mg/kg was recorded. All biomarkers were evaluated 24 hours after paracetamol injection for maximum accuracy [51].

The examination of biochemical markers showed that paracetamol significantly elevated albumin and total protein levels (Figure 1), which is probably due to its oxidative and inflammatory compartments, which can result in an increased ROS level in most tissues, especially the liver [52]. Specifically, by destroying tissue enzymes, paracetamol increases serum total protein levels [53]. Total bilirubin levels also rose after paracetamol administration (Figure 2), which like an earlier study [54] can be due to hepatocyte damage and decreased hepatic function. Furthermore, it can be said that enzyme leakage happened in the sequel to hepatocyte damage, and the lack of adequate hepatic function significantly affected the total and direct bilirubin.

In evaluating hepatic markers, like in previous studies [53, 54], the levels of ALT, ALP, and AST increased notably (Figure 3). This also can be due to severe damage to the liver and destruction of hepatocytes, which in the pathological examination, appeared in the forms of hyperemia, extensive cellular damage, and inflammation (Figure 6). After administering three doses of 125, 250, and 500 mg/kg, the greatest effect of SCE in hepatotoxic prevention was observed at a dose of 500 mg/kg, which noticeably reduced the lesions and upregulated the liver function. This pathobiological finding was in line with other biochemical results.

Moreover, in the present study, similar to the previous research [55, 56], paracetamol elevated the serum creatinine, BUN, and uric acid levels significantly (Figure 4). It seemed that after dealing with heavy damage on the nephrons and reducing the renal function in the excretion of waste products, paracetamol caused a significant rise in the levels of biochemical indices, which all were in line with the pathological findings. Following the administration of paracetamol, pathological changes were manifested as hyperemia and vast inflammation (Figure 8). So, it can be assumed that since paracetamol affected the renal function by elevating BUN levels, it can be considered a risk factor for predicting primary renal diseases in the future, which was also observed in an earlier study [56].

## 5. Conclusion

It can be concluded that paracetamol can cause toxicity in the liver and kidney tissue by damaging cells through multiple pathways. However, the findings of the current study strongly suggested that SCE can cause a dose-dependent increase in hepatorenal protection against paracetamol. Consequently, the SCE and its compounds, as a potent bioactive source for the development of better medications, could potentially be used as an antidote product, and its high tolerability would greatly reduce the risk of overdose.

## Figures and Tables

**Table 1:** The indices of vacuole degeneration, hyperemia, and infiltration of inflammatory cells in all groups in the liver.

Groups	Hyperemia	Infiltration of inflammatory cells	Vacuole degeneration
Control	—	—	—
Paracetamol	^∗∗∗^	^∗∗∗^	^∗∗∗^
SCE125 + para	^∗∗^	^∗∗^	^∗∗^
SCE250 + para	^∗∗^	^∗∗^	^∗∗^
SCE500 + para	^∗^	^∗^	^∗^
SCE1000	—	—	—

^*∗*^ *P* < 0.05 and ^*∗∗*^*P* < 0.01: significantly reduced compared to the control group; number of mice per group = 8.

**Table 2:** The indices of vacuole degeneration, infiltration of inflammatory cells, and hyperemia in all groups in the kidney.

Groups	Hyperemia	Infiltration of inflammatory cells	Vacuole degeneration
Control	—	—	—
Paracetamol	^∗∗∗^	^∗∗∗^	^∗∗∗^
SCE125 + para	^∗∗^	^∗∗^	^∗∗^
SCE250 + para	^∗∗^	^∗∗^	^∗∗^
SCE500 + para	^∗^	^∗^	^∗^
SCE1000	—	—	—

^∗^ *P* < 0.05 and ^∗∗^*P* < 0.01: significant reduction compared to the control group; number of mice per group = 8.

## Data Availability

The data used to support the findings of this study are available from the corresponding author upon request.

## Abbreviations

SCE: *Solidago canadensis* L. extract
i.p: Intraperitoneally
BUN: Blood urea nitrogen
ALT: Alanine transaminase
AST: Aspartate transaminase
ALP: Alkaline phosphatase
Para: Paracetamol
NAPQI: N-Acetyl-para-benzoquinone imine
ROS: Reactive oxygen species
PGES: Prostaglandin endoperoxide synthase.
